# Effect of Nd:YAG and Er:YAG laser tooth conditioning on the microleakage of self-adhesive resin cement

**DOI:** 10.1080/26415275.2021.1990063

**Published:** 2021-10-20

**Authors:** Azita Kaviani, Niloofar Khansari Nejad

**Affiliations:** a Department of Operative Dentistry, School of Dentistry, Ahvaz Jundishapur University of Medical Sciences, Ahvaz, Iran; b School of Dentistry, Ahvaz Jundishapur University of Medical Sciences, Ahvaz, Iran

**Keywords:** Er:YAG, laser, microleakage, Nd:YAG laser, self-adhesive resin cement

## Abstract

**Statement of problem:**

Recently, the application of lasers in restorative dentistry has been considered for cavity preparation and surface conditioning of enamel and dentin. However, the beneficial effects of cavity surface conditioning by laser irradiation on microleakage are still controversial.

**Purpose:**

This study aimed to compare the microleakage of self-etch adhesive resin cement with Nd:YAG and Er:YAG laser tooth surface conditioning to evaluate the capabilities of these lasers as a reliable replacement for etching cavities.

**Materials and methods:**

Fifty-four class V cavities were prepared on the buccal and lingual surfaces of 27 sound human premolar teeth. The samples were randomly divided into three groups (*n* = 18): group 1: no conditioning; group 2: conditioned with Er:YAG laser (2940 nm, 10 Hz, 1.2 W); group 3: conditioned with Nd:YAG laser (1064 nm, 1.5 W, 10 Hz). All the cavities were filled with self-adhesive resin cement. After curing and polishing, the samples were immersed in 2% methylene blue solution for 24 h, and after being embedded in acrylic resin, they were sectioned longitudinally and examined under a stereomicroscope. The data were submitted to Kruskal–Wallis and Dunn tests (α = 0.05).

**Results:**

The lowest microleakage mean rank was observed in the Er:YAG group (19.19), and the highest mean rank was noted in the Nd:YAG group (33.08), with significant differences between the three groups (*P*-value = .01). Pairwise comparisons demonstrated significant differences between the Er:YAG and Nd:YAG groups (*P*-value = .004) as well as Er:YAG and no conditioned groups (*P*-value =.022).

**Conclusion:**

The irradiation of the Er:YAG laser (2940 nm, 10 Hz, 1.2 W) on cavity surface resulted in less marginal microleakage of self-etch adhesive resin cement restorations compared to Nd:YAG (1064 nm, 1.5 W, 10 Hz) and no conditioning groups.

## Introduction

In bonded restorations, microleakage is a significant element [1]. Complications of microleakage in dentistry include increased saliva and microorganisms’ penetration between the restoration and tooth, secondary caries, destruction and discoloration at the margins, pulp irritation, and postoperative sensitivity that might compromise the clinical durability of the treatment. Dental caries is currently recognized as a biofilm-mediated disease, resulting from the disturbance of the homeostasis in a dynamically changing plaque biofilm, with microleakage causing more susceptibility [2,3].

Conventional resin-based cements used with etch-and-rinse adhesives used for cementing indirect tooth-colored restorations usually cause postoperative sensitivity as a common disadvantage for this cement type [4]. Opening of dentinal tubules due to acid etching has been attributed to this sensitivity [5]. The multiplicity of technical steps complicates the procedure and increases the failure rate [6]. Self-adhesive resin cements have been developed to reduce the number of steps and postoperative sensitivity [7]. Recent studies comparing the microleakage of self-adhesive resin cements to conventional resin cements have illustrated conflicting results [4,8]. According to several previous studies, the adhesion of self-adhesive resin-based luting cements to dentin and various restorative materials is satisfactory and comparable to other multi-step resin-based luting cements [9,10].

The application of lasers in restorative dentistry for surface treatment of ceramics, cavity preparation, surface conditioning, and treatment of dentin hypersensitivity has been studied extensively [11,12]. Dentin ablation with laser beams to prepare proper bonding surfaces has been introduced as an alternative method for dentin surface conditioning [6,11]. Ultra-short pulsed lasers are efficient for eliminating dental hard tissues, and their mechanism of action, referred to as ‘cold ablation’, causes even less heating [13]. The roughness produced by laser irradiation is equal to that produced by acid etching; this method was carried out using Er:YAG laser [14]. At the wavelength of 2940 nm, the energy of this laser is highly absorbed by water, which is equivalent to the amount of energy well absorbed by hydroxyapatite [15]. For this reason, the efficacy of Er:YAG laser in dentistry for caries removal, cavity preparation, and alteration of surface properties for better bonding of restorative materials to teeth has been studied by some researchers [16,17].

Nd:YAG laser is a pulsed infrared laser that is highly absorbable in pigmented tissues. This laser can be applied to tooth hard structures to increase resistance to acid attack, remineralize primary caries, alter enamel pits and fissures to prevent caries, disinfect and debride cavities, treat dentin hypersensitivity, sterilize irradiated surfaces, and increase fluoride absorption by the enamel [18]. It might also produce a glass-like appearance on the surface due to enamel and dentin heat liquefaction and re-crystallization [19]. However, the impact of laser irradiation on the surface properties of dental tissue has not been completely elucidated as to whether such irradiation can improve the surface properties of dental tissues. Laboratory studies of microleakage are often performed with the dye penetration test in class V restorations since it is a reliable, clear, and simple procedure [20,21]. Accordingly, this study aimed to compare the microleakage of self-adhesive resin cement with Er:YAG and Nd:YAG laser tooth conditioning. The null hypothesis stated that there would be no differences in microleakage score of self-adhesive resin cement after three different surface conditioning procedures: Er:YAG laser, Nd:YAG laser, nonconditioning.

## Materials and methods

The microleakage of class V cavities conditioned with Er:YAG or Nd:YAG laser and without conditioning was evaluated in this *in vitro* study. The sample size was calculated using Med Calc software with β = 0.2, α = 0.05; 18 specimens were included in each group.

Twenty-seven sound premolars with no caries, previous restorations, hypoplastic areas, and cracks, extracted for orthodontic treatment, were included in this study and stored in 0.5% sodium hypochlorite solution for 10 min after rinsing under running water. Adherent tissues were removed with a sickle scaler (Hu–Friedy Mfg. Co., Chicago, USA). The teeth were mounted in self-cured acrylic resin to facilitate cavity preparation.

**Figure 1.**
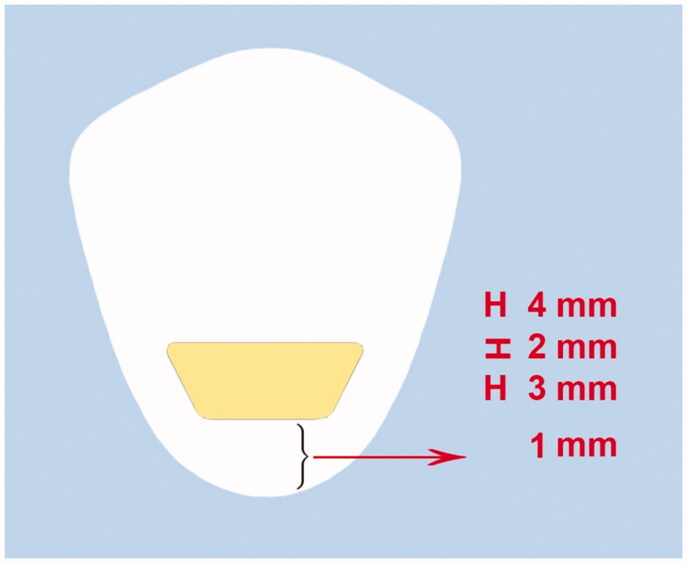
Schematic presentation of class V preparation.

Class V cavities were prepared on the buccal and lingual surfaces of each tooth using #010 fissure diamond burs (Diatech, Mt Pleasant, United States) in a high-speed handpiece (NSK, Kanuma Tochigi, Japan) cooled with air-water spray. Each bur was replaced after every five cavity preparation procedures. The preparation dimensions were 4 mm in the occlusal wall width, 3 mm in the gingival wall width, 3 mm in the occlusogingival height, and 2 mm in depth. The gingival margin was placed 1 mm coronal to the cementoenamel junction (Figure 1). Cavities were prepared by one operator, the length and width were measured using a digital caliper, and the cavities were prepared inside the drawn pattern with an inerasable pen. During preparation, the depth of the cavities was controlled with a marked periodontal probe.

The teeth were randomly divided into three groups, including 18 cavities on the buccal and lingual surfaces of teeth, for the conditioning process and restoration.

Group 1: The cavities did not undergo any surface conditioning.

Group 2: The cavities were surface-conditioned with Er:YAG laser (Fotona Light Walker, Ljubljana Slovenia, EU), with an output power of 1.2 W, a pulse repetition rate of 10 pulses/second (10 Hz), a wavelength of 2940 nm, and a pulse duration of 150 μs. The energy density was 18.9 J/cm^2^ under a continuous water mist of 5 mL/min. The HCO2-N handpiece was kept perpendicular to the dentin surface at a distance of 10 mm with a piece of orthodontic wire. The spot size of the laser beam was 0.9 mm. According to this treatment regimen, the thermomechanical ablation process might occur without producing thermal damage to the surrounding tissues, and dentin water and hydroxyapatite can absorb the maximum energy [15,22]. All the dentin surfaces were manually conditioned by one operator in vertical and horizontal directions.

Group 3: The cavities were surface-conditioned manually by one operator with Nd:YAG laser (Fotona Light Walker, Ljubljana Slovenia, EU) by a 300-mm quartz fiberoptic delivery system, and a pulse repetition rate of 10 pulses per second (10 Hz) for 15 seconds, 5 mm away from the dentin surface, using a piece of orthodontic wire, in a scanning movement. The conditioning parameters were chosen to avoid thermal damages to the tooth tissue during the thermomechanical process and to maximize energy absorption by the tissue [18,23].

All the groups were restored with self-adhesive resin cement (Embrace Wetbond, Pulpdent Corporation, Watertown, USA) (Table 1). The restorations were light-cured (LED, Woodpecker, Guilin, China) for 10 s according to the manufacturer’s instructions. The excess cement was removed with a surgical scalpel blade and polished with Sof-Lex abrasive discs (3 M ESPE Sof-Lex, Saint Paul, Minnesota, USA) from coarse to superfine. The same practitioner carried out all the procedures.

**Table 1.** Materials used in the study and their compositions.

	Manufacturer	Resin matrix	Filler content	Type	Lot Number
Embrace Wet Bond	GC, Corporation, Tokyo, Japan	Co-monomers (mono-,di-,andtri-functional methacrylatemonomers Automixsystem)	Barium,glass, ytterbiumtrifluoride,inertminerals. 36.6, 39.0%	Self-adhesive resin cement	180323

**Figure 2.**
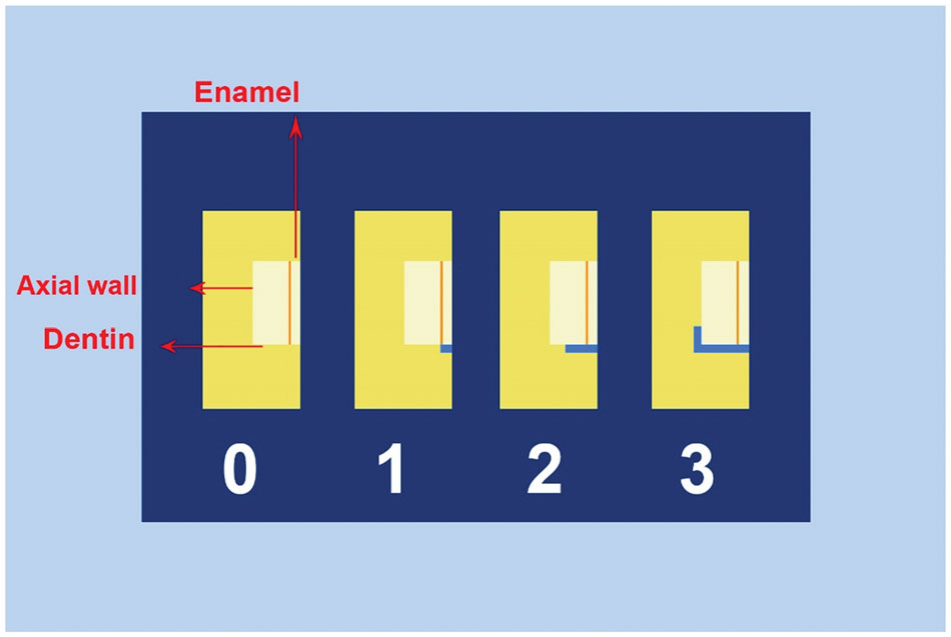
Diagram of microleakage evaluation criteria for class V cavity.

For simulating the regular temperature changes, the specimens were subjected to a 1000-cycle thermocycling procedure at 5ºC and 55 °C in a thermocycler (Rua Francisca Manoel De Oliveira, Sao Paulo, Brazil). The samples were placed in each water bath at 5ºC and 55 °C for 5 s, and a transfer time of 5 s. Next, the specimens were dried with paper. The apical foramen was closed with adhesive wax, and two coats of colored nail varnish were applied on all the surfaces of teeth (Revlon, New York, USA) except for the restoration and 1 mm short of the restoration margins to avoid dye penetration from pores and restoration margins. After the varnish dried completely, the samples were immersed in 2% methylene blue solution for 24 h and then washed to remove excess dying solution and dried [24]. The teeth were embedded in autopolymerizing transparent acrylic resin and buccolingually sectioned at the restoration center using a low-speed, water-cooled diamond saw disc (Isomet Buehler, Lake Bluff, USA). Subsequently, the dye penetration was evaluated under a stereomicroscope (Nikon, Tokyo, Japan). By only one blinded and calibrated examiner. The penetration score of methylene blue was evaluated and recorded according to criteria given in previous studies and ISO/TR 11405 standards as follows (Figure 2) [25–27]:

0: No dye penetration
1: Dye penetration through the cavity margin reaching the enamel tissue
2: Dye penetration through the cavity margin reaching the dentin tissue
3: Dye penetration through the cavity margin reaching the axial wall

Both sides were assessed, and if the scores on both sides differed, the higher score was included in the evaluation [26]. The same examiner repeated the scoring procedure. The highest score for each specimen was used to determine the final score. The data were analyzed with SPSS 22 using the Kruskal–Wallis test and Dunn test (α = 0.05).

## Results

**Figure 3.**
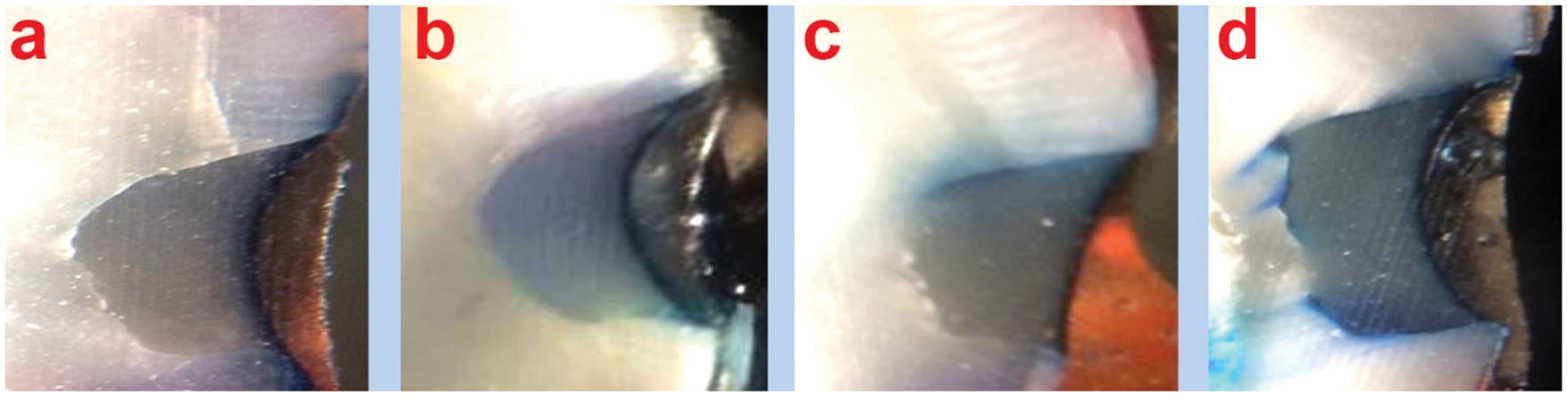
Representative stereomicroscopic images; a: no microleakage was detected (score 0, b: microleakage into the enamel portion of the cavity was detected (score 1), c: microleakage to the dentinal portion of the cavity without axial wall penetration was detected (score 2), d: microleakage into pulpal floor of the cavity was detected (score 3).

To compare the microleakage between the study groups, the data were analyzed by non-parametric Kruskal–Wallis test because the data were ordinal. Table 2 presents the microleakage scores in the study groups. Figure 3 shows microscopic images of various scores of dye penetration. The mean rank of microleakage scores of nonconditioned samples as the control group, Er:YAG group, and Nd:YAG group were 30.22, 19.19, and 33.08, respectively. The results demonstrated a significant difference between the three groups (*p* = .010). Pairwise comparisons are illustrated in Table 3. According to the result of the Dunn test, there were significant differences in the marginal microleakage scores between the Er:YAG laser group and the Nd:YAG laser group (*P*-value = .004) and also the Er:YAG laser group and the non-conditioning group (*P*-value = .022). No significant difference was found between the Nd:YAG laser group and the unconditioned cavities (*P*-value = .553) (Table 3).

**Table 2.** Comparison of marginal microleakage scores in the study groups by Kruskal–Wallis test.

	Microleakage				Total	Mean Rank	Test statistic (P-value)
	0	1	2	3			
Groups							
Control							
Count	5	11	2	0	18	30.22	9.26 (.010)
% within groups	27.8%	61.1%	11.1%	.0%	100.0%		
Er:YAG							
Count	13	4	1	0	18	19.19	
					
% within groups	72.2%	22.2%	5.6%	.0%	100.0%		
Nd:YAG							
Count	5	8	3	2	18	33.08	
% within groups	27.8%	44.4%	16.7%	11.1%	100.0%		
Total							
Count	23	23	6	2	54		
% within groups	42.6%	42.6%	11.1%	3.7%	100.0%		

**Table 3.** Pairwise comparisons of microleakage between the groups by the Dunn test.

	Test statistic	*p* Value
Control: Er:YAG	11.03	.022
Er:YAG: Nd:YAG	−13.89	.004
Control: Nd:YAG	−2.86	.553

## Discussion

Dental caries is currently recognized as a biofilm-mediated disease, resulting from the disturbance of the homeostasis in a dynamically changing plaque. Microleakage is one of the most important causes of these disruptions, which is defined as the accumulation of bacterial fluids, chemicals, and ions between the cavity walls and the restorative materials that is not clinically detectable [28].

This study compared the microleakage of self-adhesive resin cement with Er:YAG (2.94 μm, 10 Hz, 1.2 W, 18.9 J/cm^2^) and Nd:YAG laser tooth conditioning. The results illustrated that surface conditioning with Er:YAG (2940 nm, 10 Hz, 1.2 W, 18.9 J/cm^2^) laser can significantly decrease microleakage between dentin and self-adhesive resin-based luting cement compared with the nonconditioned surface and the Nd:YAG group. Therefore, the null hypothesis was partially rejected.

The commonly used lasers in dentistry are the Nd:YAG laser with 1064 nm and erbium lasers with 2940 nm wavelength. In these wavelengths, lasers have higher absorption, less penetration depth, and fewer thermal side effects since the energy is absorbed by the tissue absorption mechanism and its associated processes [29]. The water and mineral content and the laser’s energy, power, and wavelength are parameters that lead to different reactions with enamel, dentin, and cementum. The parameters in this study were selected based on previous studies on the morphology and temperature of dentin exposed to leaser irradiations with different intensities and frequencies to achieve the best results in terms of the lowest heat produced in the substrate, low pulpal irritation, and the best bond between the adhesive and tooth structure. Since the 2940-mm wavelength of Er:YAG laser exhibits high absorption in water, it heats the substrate. Therefore, it is necessary to use air-and-water coolant to prevent pulpal damage [15,18,30].

Based on the results of the present study, the groups without conditioning exhibited the highest microleakage; however, the difference was significant only from the Er:YAG laser group. The self-adhesive resin cement used in the present study was Embrace Wetbond, which contains mono-, di-, and tri-functional methacrylate monomers and a resin acid integrating network that is activated in the presence of moisture, resulting in the simultaneous demineralization and penetration of the hydrophilic monomer into demineralized dentin. As illustrated in previous studies [31,32], one of the drawbacks of self-etch adhesives is a lack of complete penetration into the smear layer and dissolution of the smear plug. Therefore, a lack of the complete ability of the acid in changing the dentin substrate and preparing it for the penetration of the adhesive into dentin might be the reason for a possibly high mean microleakage in the group without preparation in the present study.

In the present study, the best results concerning decreased microleakage were achieved in the Er:YAG laser group, consistent with a study by Molds *et al*, in which the microleakage of Cl V composite resin restorations irradiated with Er:YAG and Er,Cr:YSSG in association with self-etch or etch-and-rinse adhesive systems were evaluated [33]. It was concluded that using self-etch adhesives in cavities that prepared with Er:YAG and Er,Cr:YSSG effectively decreased microleakage. In the study by Molds *et al*, 2940-nm Er:YAG laser with 89.1 J/cm^2^ in enamel and 76.4 J/cm^2^ energy in dentin with 10 Hz frequency was applied [33]. Although Er:YAG laser with 18.9 J/cm^2^ energy was used in the present study for conditioning, the results of the two studies were consistent. Therefore, it seems that the laser energy in the present study was adequate to change the organic components of dentin and prepare it for the boding of the self-etch resin cement.

According to Moldes *et al.*, this effect can be attributed to the structure of the enamel and dentin produced by Er:YAG laser ablation, which causes the formation of mechanical retentive patterns, removal of the smear layer, and changes in morphology and possibly chemical changes in the minerals and organic content of tooth hard structures. After laser irradiation, there would be a better interaction and chemical bonding between the acidic resin monomers with the residues of the products remaining after the laser process [33,34]. Er:YAG laser irradiation leads to the sudden evaporation of the water content of the tooth hard tissue, increasing the internal pressure of dentinal tubules. This strain disrupts the mineral content of the hard tissue until it is melted by laser irradiation, resulting in surface roughness in both the macroscopic and microscopic measurements. The superficial bond of self-adhesive cement with dentin causes partial demineralization of the smear layer, leading to the formation of short resin tags [6]. Since a rugged and porous surface provides a greater surface area for bonding and making deeper resin tags, which results in higher bond strength and lower microleakage, this visible difference in surface roughness can significantly affect the microleakage or bond strength of restorative materials. The laser technique might also work acceptable because it performs the etching without damaging the underlying tissues and the tooth pulp [35,36]. Similar to the results of the present study, Hossain *et al.* reported that using an Er:YAG laser (2.94 m, 50.9 J/cm2, 2 Hz) for cavity preparation might decrease microleakage of composite resin restorations [26]. According to the results of this research, the cavities irradiated with Er:YAG laser have a rough surface, prominent prismatic plates in enamel, exposed and open dentinal tubules, and scaly and irregular appearance in dentin due to laser beam ablation. In this way, microleakage decreases with an increased adhesive surface [26].

Contrary to the present study, Ramos *et al* reported that Er:YAG laser (19.3 J/cm^2^, 0.12 W, 2 Hz, 60 mJ, and 2.94 µm) for the conditioning of dentin before the application of Clearfil SE Bond self-etch adhesive did not result in a significant difference from the group without conditioning, reporting that possibly the 10-MDP component in the self-etch adhesive have adequate strength to chemically bond the chemical components of the adhesive to the residual calcium ions of hydroxyapatite. Therefore, there was no significant difference from the bond strength in the group conditioned with Er:YAG laser [37]. However, in the present study, the Embrace Wetbond resin cement did not contain 10-MDP. In addition, the frequency and power of the laser were different from the study above, which might explain the differences in the results of the present study.

Esteves–Oliviera *et al* utilized Er:YAG (2.94 μm, 60 mJ, 2 Hz, 0.12 W, 19.3 J/cm2 ) for pretreatment of cavities prepared with Er:YAG laser (400 mJ/2 Hz) and evaluated microleakage of CL V cavities restored using a self-etch adhesive, reporting no need for additional conditioning of these cavities with weaker Er:YAG laser. They suggested that self-etch adhesives are a proper choice for restoring cavities prepared with Er:YAG laser because one of the drawbacks of these self-etch adhesives is the inadequate etching of the thick smear layer. In addition, in cavities prepared with Er:YAG laser when the external surface of dentin is devoid of the smear layer and smear plug, allowing adequate penetration of the smear layer and formation of more extended micro tags [38]. In the present study, pretreatment with Er:YAG laser resulted in minimum microleakage.

In contrast to the present study, Bastos Ramos *et al* evaluated the effect of cavity preparation with Er:YAG (2:94 nm wavelength) with two energy/pulse levels of 250 mJ/2 Hz and 400 mJ/2 Hz on the tensile bond strength of two types of adhesives (self-etch and total etch) to enamel and dentin. They reported that cavity preparation with this laser type did not affect the adhesive properties of these agents compared to the conventional techniques. However, they suggested that Er:YAG laser can be a choice comparable to conventional techniques because considering the deeper penetration of the adhesive into the patent dentinal tubules after laser preparation, a better marginal seal will probably be achieved [38]. In the present study, preparation with Er:YAG laser resulted in a significant decrease in the microleakage of the self-adhesive resin cement compared to Nd:YAG laser (10 Hz, 1.2 W, 1064 nm) and the group without pretreatment.

In the present study, there was no significant difference in the microleakage between Nd:YAG laser restorations and non-lased restorations (*P*-value = .553). Therefore, it seems that Nd:YAG (1064, 1.2 W, 10 Hz) laser with the parameters used in this study cannot create the predicted tooth surface change. Consistent with the present study, Acar *et al* evaluated the effect of preparation with Nd:YAG laser with 1 W power and 15 Hz frequency on the tensile bond strength of self-adhesive resin cement to dentin. The results showed that this variable was not significantly different between the laser and non-conditioning groups, which might be attributed to the occlusion of dentinal tubules after irradiation with Nd:YAG laser beams and prevention of cement penetration into the dentinal tubules. In the present study, although the laser parameters were different (1.5 W/10 Hz), similar results were achieved [39].

Despite the present study, Wax *et al* evaluated the effect of different power and frequencies of Nd:YAG laser on the bond strength and microleakage of class V cavities. they reported the highest bond strength with 1 -W power and 15 Hz frequency, which was higher than that in the group conditioned with 35% phosphoric acid [40]. In the present study, 1.5 W power and 10 Hz frequency were used with self-etch resin cement. Although the Nd:YAG laser group had a greater mean rank of microleakage than the nonconditioning group, the difference was not significant. Wax *et al.* believed that the removal of the smear layer and surface roughness following melting and re-crystallization of the dentin surface by Nd:YAG laser irradiation, sealing the dentinal tubule orifices, and strengthening of the hybrid layer due to the residual integration of the smear layer with the intact dentin are the causes of this phenomenon [40].

It should be noted that the two variables of energy and frequency affect the interaction of the Nd:YAG laser with the dentin surface. Moritz *et al.* demonstrated that a setting of 1.5 W for Nd:YAG laser has the best results in terms of bactericidal activity with less risk of thermal tissue damage [29]. Excessively low output energy does not affect the dentin surface, whereas excessive output energy decreases surface roughness, resulting in decreased dentin adhesion. The frequency which determines the energy per pulse affects the depth of the dentin surface irradiated and the thickness of the hybrid layer formed. Therefore, the dentin adhesion is highly affected by the appropriate energy and frequency of Nd:YAG laser [12].

Aranha *et al* investigated the impact of Er:YAG and Nd:YAG lasers on the permeability of composite resin restorations on root dentin in a study similar to this one and found that Er:YAG and Nd:YAG lasers were both effective at reducing dentin permeability, with no statistical differences. They claimed that because of its high wavelength absorption by water and partial degradation of dentinal tubules, the Er:YAG laser induces evaporation of dentinal fluid and the smear layer, reducing dentin permeability [41], almost similar to the current study’s findings. This finding contradicts the results of the present study, and it seems that failure to use proper laser parameters is the reason for this inconsistency.

The interactions between different resin-bonding monomers with laser-treated dental tissues have been the focus of various studies, demonstrating that the interaction mechanism is not well-defined, and further research is needed on laser energy, adsorption, and interaction with hydroxyapatite, collagen fibers, and water present in hard tissues [33,42]. Then, more experiments and studies are required to measure the bond strength of resin cement to the tooth structure and scan with a scanning electron microscope (SEM for accurate observation of changes. It is also necessary to use other laser parameters for closer examinations in clinical conditions.

## Conclusion

Under the limitations of this *in vitro* study and the parameters selected for surface conditioning, it can be concluded that using Er:YAG for the surface conditioning of cavities restored with self-adhesive resin cement resulted in more microleakage reduction than using Nd:YAG laser for surface conditioning or no conditioning. To verify these findings, more research with larger sample size or alternative laser parameters, as well as clinical assessments, are essential.
